# Effectiveness of mobile oral health intervention on orthodontic patients’ oral hygiene and oral health literacy: a randomised controlled clinical trial

**DOI:** 10.1186/s12903-025-07423-y

**Published:** 2025-12-12

**Authors:** Nermine M. Balbaa, Maha A. Hamza, Wafaa E. Abdelaziz, May M. Adham

**Affiliations:** Department of Paediatric Dentistry and Dental Public Health, Faculty of Dentistry, Alexandria University, Champolion St, Azarita, Alexandria 21527 Egypt

**Keywords:** Oral health literacy, Oral hygiene, Mobile oral health intervention, Orthodontic patients, OHL-AQ, Plaque index, WhatsApp messages

## Abstract

**Background:**

Fixed orthodontic appliances pose challenges to maintaining oral hygiene by increasing plaque retention, leading to gingival inflammation. Oral Health Literacy (OHL) is essential for understanding and implementing oral hygiene instructions effectively. Mobile oral health interventions, including smartphone-based programmes, have recently emerged as promising tools to improve oral hygiene behaviours and outcomes. This trial assessed the effectiveness of the World Health Organisation (WHO) mOralHealth programme in improving orthodontic patients’ oral hygiene and OHL.

**Methods:**

A randomised controlled trial was conducted involving 60 orthodontic patients aged 16–25 in the orthodontic clinic of Faculty of Dentistry, Alexandria University, Egypt. Oral hygiene was measured using the Silness-Löe Plaque Index (PI). OHL was assessed using the validated Arabic version of the Oral Health Literacy Adult Questionnaire (OHL-AQ) across four domains. Baseline assessments (T0) for both OHL and PI were conducted before randomly assigning participants to either a control group (*n* = 30), receiving standard oral hygiene instructions, or an intervention group (*n* = 30), receiving the same instructions in addition to the WHO mOralHealth programme (46 WhatsApp messages delivered over 12 weeks). OHL and PI were reassessed after one month (T1) and three months (T2) and compared to T0 to monitor changes in both outcomes. Data collected were analysed using SPSS. The association between PI and OHL was assessed at all time points.

**Results:**

At T0, no significant differences were observed between groups in demographics, oral hygiene behaviours, or OHL. At T2, the intervention group showed significantly lower median PI scores (1.33, IQR = 1.00-1.63) than the control group (1.83, IQR = 1.50-2.00; *p* = 0.001). OHL scores were also significantly higher in the intervention group, particularly in the reading and numeracy domains (*p* < 0.01). Plaque scores were negatively correlated with numeracy (*p* = 0.012) and decision-making (*p* = 0.045) within the intervention group.

**Conclusion:**

The mOralHealth programme implemented via WhatsApp significantly improved oral hygiene and OHL among orthodontic patients over three months compared to standard oral hygiene instructions, with potential for integration into orthodontic care as a practical and scalable approach to enhance plaque control, sustain oral hygiene habits, and support long-term behavioural change when reinforced over time.

**Trial registration:**

ClinicalTrials.gov, TRN: NCT06734325, Registration date:11 December 2024, retrospectively registered.

## Introduction

The World Health Organisation (WHO) estimated, in March 2025, that approximately 3.7 billion people are affected by oral diseases, compared to the 3.5 billion reported in the Global Oral Health Status Report, in 2022 [1]. This apparent increase may be attributed to improved surveillance and enhanced data reporting methods, and global population growth, rather than a true deterioration in oral health status [2]. Severe periodontal disease and untreated dental caries, in both deciduous and permanent teeth, were listed amongst the major contributors to the global burden of oral disease [1]. Despite the importance of adopting appropriate, regular and optimal oral health promoting behaviours for preventing dental diseases, many people still lack fundamental oral health information, education, and knowledge on how to perform and maintain appropriate oral hygiene [3–5].

Health literacy (HL) is defined as “the degree to which individuals have the capacity to obtain, process, and understand basic health information and services needed to make appropriate health decisions” [6]. Research has indicated that HL significantly influences an individual’s health, health-related behaviours, general health outcomes, and utilisation of healthcare [7]. Meanwhile, inadequate health literacy is identified as a root cause of health inequalities and is now recognised as a national health priority in many countries [8, 9].

Oral health literacy (OHL), on the other hand, is a subset of the broad concept of health literacy and has recently gained increased attention in dental literature [10, 11]. It is defined as “the degree to which individuals have the capacity to obtain, process, and understand basic oral health information and services needed to make appropriate health decisions” [12]. OHL involves competency in reading, writing, counting, speaking, listening, and proper decision-making. According to the conceptual model by Macek et al. [13], health outcomes are the result of their health-related decisions, which are modulated by health literacy that are influenced by several sociodemographic factors, such as income, education, and personal characteristics. While HL refers to an individual’s ability to obtain, understand, and use health information, OHL extends these skills to specific oral contexts such as interpreting fluoride recommendations, understanding toothbrushing techniques, and following orthodontic care instructions. Similar to HL, OHL skills are essential for encouraging individuals to improve their oral health and reduce disparities [11]. Low OHL is a significant contributing factor to the high prevalence of oral diseases and failure to adopt behaviours that have been shown to help maintain health, particularly among patients undergoing complex dental treatments like orthodontic treatment [14]. Baskaradoss [15] showed that individuals with low OHL levels are most likely to have missed dental appointments, have a higher prevalence of dental caries, poorer periodontal conditions, and more missing teeth as a result of improper oral health behaviours and using dental services for emergencies only [14, 16–18].

Having adequate oral health knowledge and self-efficacy is essential for adopting healthy practices and changing behaviours. Previous studies have shown that increased knowledge of oral health is highly correlated with better oral hygiene and health-related behaviours [19–22].

Orthodontic patients, particularly those undergoing fixed orthodontic treatment, face unique challenges in maintaining optimal oral hygiene due to the complexity of orthodontic appliances, which can serve as reservoirs for plaque accumulation around orthodontic brackets and wires [23, 24] increasing the risk of gingival inflammation and the development of White Spot Lesions (WSLs) [25, 26]. Understanding the role of OHL in shaping oral hygiene behaviours among orthodontic patients is essential for improving treatment outcomes and promoting long-term oral health. Patients undergoing fixed orthodontic treatment with higher levels of OHL may be more proficient in overcoming the oral hygiene challenges associated with orthodontic treatment by acquiring preventive behaviours such as regular brushing, flossing, and dental check-ups, leading to improved oral health outcomes and treatment success [27, 28].

Digital-enabled interventions are gaining momentum as internet access, smartphone usage, and digital literacy continue to increase among developed and developing nations [29]. Given that individuals with limited OHL often poorly engage with traditional health educational methods, the integration of novel approaches, such as the use of mobile health (mHealth) technologies like WhatsApp reminders may provide a more engaging and accessible opportunity for improving oral health knowledge and behaviours and supporting patients in maintaining optimal oral hygiene throughout their orthodontic treatment [30]. The mOralHealth programme, a joint initiative by the WHO and the International Telecommunication Union (ITU), aims to promote the use of mobile technologies to address the burden of Non-Communicable Diseases (NCDs), including oral diseases [31]. However, there is insufficient evidence to support the effectiveness of such mobile oral health interventions in improving oral hygiene outcomes and OHL among orthodontic patients, particularly in Arabic-speaking populations. Therefore, this study aimed to (1) assess the effectiveness of the mOralHealth programme implemented via WhatsApp messages as compared to standard oral hygiene instructions in improving oral hygiene and OHL levels among orthodontic patients after three months and (2) determine the association between oral hygiene and OHL levels. The null hypothesis was that the mOralHealth programme would have no significant effect on either oral hygiene or OHL.

## Materials and methods

### Study design and setting

This Randomised controlled clinical trial (RCT) with two parallel groups was conducted in accordance with the Consolidated Standards of Reporting Trials (CONSORT) guidelines [32] [see Additional file 1]. It was retrospectively registered in ClinicalTrials.gov (NCT06734325) on December 11th 2024 and approved by the Research Ethics Committee, Faculty of Dentistry, Alexandria University (# 0913-05/2024). The trial took place in the orthodontic clinic in the Faculty of Dentistry, Alexandria University, Egypt, from December 2024 to March 2025. Data were collected at: baseline (T0), one month follow-up (T1) and three months follow-up (T2). All assessments were performed before patients entered the clinic to avoid potential influence of professional manipulation on oral hygiene.

### PICO question

For patients undergoing orthodontic treatment with fixed appliances (P), does the implementation of mobile oral health intervention (I) as compared to standard oral hygiene instructions (C) result in an improvement in oral hygiene and OHL (O)?

### Sample size

The sample size was estimated based on assuming a 95% confidence level and 80% study power. The mean Plaque Index score after 6 months was 1.82 ± 0.78 for the standard oral hygiene instructions and 1.25 ± 0.76 for the mobile oral health intervention group [33]. Based on the difference between the two independent means, a sample of 24 patients per group was required, yielding an effect size of 0.740. Total sample size = number per group × number of groups = 24 × 2 = 48 patients. To address potential losses to follow-up, the sample size was increased to 60 participants.

### Software

The sample size was determined using Rosner’s method [34], as calculated by G*Power 3.0.10 [35].

### Eligibility criteria

Patients were included if they were undergoing orthodontic treatment with fixed appliances in both arches for the first time, between the ages of 16 and 25 years, owned a smartphone with WhatsApp installed, provided informed consent and agreed to participate. Exclusion criteria included the presence of systemic diseases affecting oral health, patients with cleft lip and/or palate, functional and orthognathic patients, those who had undergone previous orthodontic treatment, and patients with a removable appliance [36].

### Randomisation and allocation concealment

Participants were randomly allocated into intervention and control groups in a 1:1 ratio using a computer-generated random numbers list [37]. Allocation concealment was achieved through sequentially numbered, opaque, sealed envelopes having the name of the patient prepared by a dental assistant who was not involved in the trial. Each envelope was only opened at the time of intervention [38].

### Blinding

Due to the nature of the intervention, blinding of participants and the intervention provider was not possible.

### Intervention

All participants received a clear explanation of the trial aim, intervention, methods, and timelines before starting the intervention. Participants were randomly assigned to one of two groups:

#### Control group

 Participants received standard oral hygiene instructions during follow-up visits in accordance with those from their orthodontists, including a demonstration on dental models for brushing after each meal using a circular motion and how to use interdental brushes [39].

#### Intervention group

 Participants received the same instructions as the control group in addition to the mOralHealth programme [31], designed to align closely with the constructs of the Health Belief Model (HBM) [40], which emphasise that individuals’ engagement in preventive behaviours depends on their perceived susceptibility to disease, perceived benefits of action, cues to action and self-efficacy. The programme delivered 46 WhatsApp messages (Appendix I) over 12 weeks, beginning with daily messages (weeks 1–4) and gradually decreasing to three times weekly (weeks 5–8), then twice weekly (weeks 9–10), and once weekly (weeks 11–12). Higher initial frequency served as cues to action to establish behaviour, repeatedly reminding participants of their susceptibility to oral diseases and the severity of potential outcomes such as caries or gingival inflammation if preventive care is neglected, emphasizing the benefits of proper oral hygiene practices, such as plaque control and improved aesthetics. The gradual reduction in message frequency encouraged the development of self-efficacy, enabling participants to maintain new behaviours independently, aligning with the concept that consistent reinforcement over time helps consolidate habits into routine practice. Messages were culturally adapted by forward-translating into Arabic, then back-translating, comparing the result with the original text to identify any differences and reviewing for contextual appropriateness by bilingual experts to ensure cultural relevance and ease of understanding for the target population. All patients in the intervention group received the same sequence of messages, starting with informational content, followed by motivational messages, and concluding with reminders to reinforce oral hygiene practices. All interactions with patients, including data collection and the messages, were conducted in Arabic. Participant engagement with WhatsApp messages was actively tracked through read receipts, interactions and replies.

### Follow-up

The follow-up assessment was conducted one (T1) and three (T2) months after baseline, aligning with the 12-week duration of the mHealth intervention. This period was considered appropriate to assess the short-term effects of knowledge acquisition and enhanced OHL on oral hygiene behaviour. The three-month follow-up aligns with the expected consolidation period for behavioural change, allowing sufficient time for knowledge acquisition and improved OHL to translate into measurable hygiene outcomes. Extending the follow-up duration could have increased attrition without substantially changing short-term behavioural outcomes.

### Outcomes assessment

Participants’ demographic data, self-reported oral health, toothbrushing frequency, and use of fluoridated toothpaste were collected using the Arabic version of WHO’s oral health assessment questionnaire for adults [41] at T0.

I. Assessment of oral hygiene level
  The Silness-Löe Plaque Index (PI) [42], developed in 1967, was used to assess participants’ oral hygiene at T0 before randomisation. The presence of dental plaque was evaluated in four surfaces, namely mesiobuccal, buccal, distobuccal, and lingual of six index teeth #16, 12, 24, 36, 32, and 44, and each surface was given a score of 0 to 3 [see Additional file 2]. The four surfaces of each tooth were scored from 0 to 3, and scores were averaged per tooth and then per individual. PI was measured again at T1 and T2, and scores were compared to T0 to monitor changes in participants’ PI scores after the intervention. The outcome assessor responsible for measuring the PI was trained and calibrated prior to data collection to ensure consistency and reliability in scoring.
II. OHL assessment
  The OHL level of all participants was assessed at T0 prior to randomisation using the validated Arabic Version of the Oral Health Literacy—Adult Questionnaire (OHL-AQ) [43], which consisted of 17 questions, divided into four domains— Reading, Numeracy skills, Decision-making, and Listening:
  1. The *reading comprehension* section consisted of three questions with six missing words or phrases, designed to assess participants’ awareness of oral diseases and knowledge of oral health.
  2. In the *numeracy skills* section, participants were presented with two statements about prescriptions for antibiotics and mouth rinse and then asked four questions to assess their understanding and calculation skills.
  3. The *decision-making* section consisted of five multiple-choice questions related to common dental problems and information from medical history, assessing patients’ decision-making skills regarding common oral health problems.
  4. In the *listening* section, patients heard post-extraction instructions and were asked two questions to assess their listening and communication skills.
    Participants were allowed to fill out the form only once with no time restriction. Each question in the questionnaire had one correct answer and one wrong answer or ‘I don’t know’. Each correct answer was scored as “1” and incorrect or ‘I don’t know’ answers were scored as “0”. Scores of all questions answered by participants were added to obtain the overall score of OHL ranging from 0 to 17 [44]. Scores at T1 and T2 were compared with T0 to monitor the change in participants’ OHL level after receiving oral hygiene instructions and the intervention.
III. Association between PI and OHL

### Data management and statistical analysis

Data were analysed using IBM SPSS Statistics for Windows version 26.0 (IBM Corp., Armonk, NY, USA). Descriptive statistics were used to summarise baseline characteristics. Normality of continuous variables was assessed using the Kolmogorov–Smirnov test. As the test indicated non-normal distribution, non-parametric tests were used for group comparisons. The Mann–Whitney U test was used to compare PI and OHL scores between the intervention and control groups at each time point. Subgroup analyses were conducted to examine differences in individual OHL domains (reading comprehension, numeracy, listening, and decision-making). A *p*-value of < 0.05 was considered statistically significant. To explore factors influencing oral health outcomes beyond simple between-group differences, multivariable linear regression analyses were performed to identify predictors of PI and OHL scores at the three-month follow-up. Effect sizes were calculated for all comparisons, and 95% confidence intervals (CI) were reported. Effect sizes were interpreted according to physiotherapy research standards [45], given the comparable functional context of the stomatognathic system. Missing data were handled using the last observation carried forward.

### Benefits/harms

Benefits from the study included: (1) Improved oral hygiene practices as a result of receiving targeted interventions and (2) Increased awareness of OHL, encouraging individuals to actively engage in better oral hygiene practices. There were no anticipated or observed harms associated with participation in this trial. The only potential inconvenience was the time required from participants to complete the OHL questionnair

## Results

**Fig. 1:**
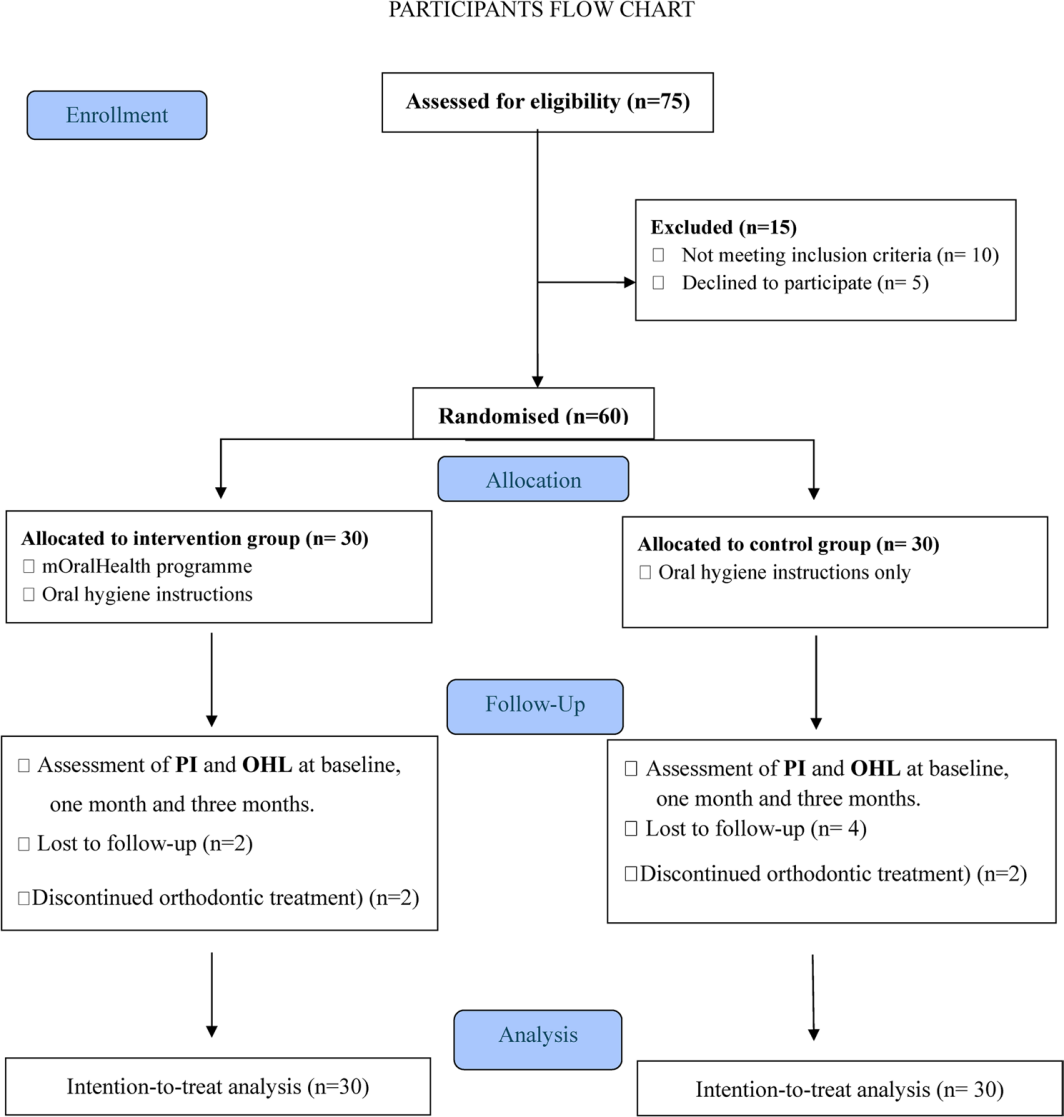
Flow chart showing the flow of participants from screening for eligibility to follow-up assessments. A total of 75 orthodontic patients were assessed for eligibility, of whom 60 met the inclusion criteria and were randomly allocated into two equal groups: intervention (n=30) and control (n=30). All participants received the assigned interventions, and follow-up assessments were conducted at one and three months. Two participants in the intervention group were lost to follow-up and two had discontinued their orthodontic treatment. In the control group, four participants were lost to follow-up and two had discontinued their orthodontic treatment. Intention-to-treat analysis was used, with all randomised participants included in the analysis. Missing data were handled using the last observation carried forward

Of the 60 participants randomised, 50 participants (83%) completed the 3-month follow-up. Four participants discontinued orthodontic treatment after one month and six were lost to follow-up. However, all randomised participants were included in the final analysis (Fig. 1).

The baseline demographic characteristics of the intervention and control groups were comparable, with no statistically significant differences observed across the assessed variables (Table 1). The mean age was slightly lower in the intervention group (18.07 ± 2.35 years) compared to the control group (18.90 ± 2.31 years). The proportion of female participants was higher in the intervention group (76.7%) than in the control group (56.7%). Educational levels were similarly distributed across both groups, with high school being the most reported level.

**Table 1:** Baseline demographic characteristics of participants

		Control Group (*n* = 30)	Intervention Group (*n* = 30)	*p* value
		Mean ± SD	Mean ± SD	
Age (years)		18.90 ± 2.31	18.07 ± 2.35	0.120
		n(%)	n (%)	
Gender	Males	13(43.3%)	7(23.3%)	0.100
	Females	17(56.7%)	23(76.7%)	
Education	Primary	2(6.7%)	2(6.7%)	0.958
	Middle	13(43.3%)	11(36.7%)	
	High school	13(43.3%)	15(50%)	
	University	2(6.7%)	2(6.7%)	
Pain in the last 12 months	No	23(76.7%)	23(76.7%)	1.000
	Yes	7(23.3%)	7(23.3%)	
Toothbrushing frequency	2–6 times/week	0	1(3.3%)	0.601
	Once daily	4(13.3%)	4(13.3%)	
	Twice daily	26(86.7%)	25(83.3)	
Toothbrush use	No	0	0	-
	Yes	30(100%)	30(100%)	
Dental floss use	No	30(100%)	26(86.7%)	0.112
	Yes	0	4(13.3%)	
Interdental brush use	No	19(63.3%)	19(63.3%)	1.000
	Yes	11(36.7%)	11(36.7%)	
Toothpaste use	No	0	0	-
	Yes	30(100%)	30(100%)	
Previous Dental visit	No	12(40%)	12(40%)	1.000
	Yes	18(60%)	18(60%)	

*Statistically significant differences at *p* value < 0.05

Pain experience during the previous 12 months was identical between groups, with 76.7% in each group reporting no pain. Most participants (85%) reported brushing twice daily, and all reported using a toothbrush and toothpaste. Dental floss use was reported by 13.3% in the intervention group while none in the control group reported using it. Use of interdental brushes and history of dental visits were consistent between the two groups. Participants’ engagement with the messages was high, with 93.3% of participants viewing at least 80% of the messages.

### I-Oral hygiene

As shown in Table 2, the control group showed a deterioration in oral hygiene, reflected by an increase in the median PI score from 1.50 (IQR = 0.71) at T0 to 1.58 (IQR = 0.50) at T1 and 1.83 (IQR = 1.50–2.00.50.00) at T2. In contrast, the intervention group demonstrated improved oral hygiene over time, with a decrease in the median PI scores from 1.67 (IQR = 1.00) at T0 to 1.58 (IQR = 0.88) at T1 and 1.33 (IQR = 1.00–1.63) at T2. No significant differences in PI scores were found between the two groups at baseline (*p =* 0.727) or at the one-month follow-up (*p =* 0.543), confirmed by the 95% confidence intervals crossing zero. Although a small improvement was seen in the intervention group at T1 (Cohen’s d = 0.252), it was neither statistically nor clinically significant. However, at T2, the CI excluded zero, showing a statistically significant (*p =* 0.001) and clinically meaningful improvement in the intervention group, with a large effect size (Cohen’s d = 0.869). Within-group comparisons revealed a significant improvement in the intervention group from baseline to three months (*p* < 0.001), while no significant changes were observed in the control group over time.

**Table 2:** Comparison of plaque index between intervention and control groups at different follow-up intervals

		Control Group (*n* = 30)	Intervention Group (*n* = 30)	95% Confidence Interval (CI)	Cohen’s d	*p* value
Baseline (T0)	Mean ± SD Median(IQR)	1.69 ± 0.56 1.50(0.71)	1.72 ± 0.54 1.67(1.00)	−0.23 to 0.17	0.055	0.727
1 month (T1)	Mean ± SD Median(IQR)	1.75 ± 0.54 1.58(0.50)	1.62 ± 0.49 1.58(0.88)	−0.13 to 0.39	0.252	0.543
3 months (T2)	Mean ± SD Median(IQR)	1.86 ± 0.50 1.83(1.50–2.00.50.00)	1.43 ± 0.49 1.33(1.00–1.63.00.63)	0.17 to 0.69	0.869	0.001*

Within-group comparisons over time were significant for both control (*p = 0.012*) and intervention groups (*p < 0.001*)

Pairwise post-hoc results: control (*p₁=1.000*, *p₂=0.051*, *p₃=0.280*); intervention (*p₁=0.060*, p₂<0.001*, *p₃=0.244*)

*Statistically significant differences at *p* value < 0.05, p₁: comparison between baseline and 1 month, p₂: comparison between baseline and 3 months, p₃: comparison between 1 month and 3 months

### II-Oral health literacy

As shown in Table 3, there were no significant differences between the intervention and control groups across any domain of OHL at T0 (*p* > 0.05). Both groups had a median reading score of 3.00 (IQR = 2.00, *p =* 0.902). Small effect size together with baseline CI crossing zero further confirmed group comparability. However, at T1 and T2, the intervention group showed significantly higher reading scores than the control group (*p =* 0.001 and *p =* 0.002, respectively) with large effect sizes and 95% confidence intervals excluding zero. Within-group analysis revealed significant improvements in reading overtime in the intervention group (*p* < 0.001), with notable increases from T0 to T1 (*p =* 0.024) and from T0 to T2 (*p =* 0.001). No significant change was found between T1 and T2 (*p =* 0.817). The control group did not exhibit significant changes over time in reading (*p =* 0.107).

**Table 3:** Comparison of OHL between intervention and control groups at different follow-up intervals

			Control Group (*n* = 30)	Intervention Group (*n* = 30)	95% Confidence Interval (CI)	Cohen’s d	*p* value
Reading	Baseline (T0)	Mean ± SD Median(IQR)	2.90 ± 1.42 3.00(2.00)	2.87 ± 0.97 3.00(2.00)	−0.60, 0.66	0.025	0.902
	1 month (T1)	Mean ± SD Median(IQR)	2.87 ± 1.33 3.00(1.25)	3.87 ± 0.97 4.00(2.00)	−1.60, −0.40	0.860	0.001*
	3 months (T2)	Mean ± SD Median(IQR)	3.13 ± 1.31 3(1.25)	4.13 ± 0.97 4.00(2.00)	−1.60, −0.40	0.868	0.002*
Numeracy	Baseline (T0)	Mean ± SD Median(IQR)	2.5 ± 1.22 2.00(2.25)	2.97 ± 1.21 3.00(2.00)	−1.10, 0.16	0.387	0.110
	1 month (T1)	Mean ± SD Median(IQR)	2.60 ± 1.22 3.00(2.25)	3.40 ± 1.03 4.00(1.00)	−1.38, −0.22	0.709	0.004*
	3 months (T2)	Mean ± SD Median(IQR)	3.00 ± 1.11 3.00(1.25)	3.40 ± 0.97 4.00(1.00)	−0.94, 0.14	0.384	0.095
Decision-making	Baseline (T0)	Mean ± SD Median(IQR)	3.13 ± 1.28 3.00(2.00)	2.93 ± 1.46 3.00(2.25)	−0.51, 0.91	0.146	0.581
	1 month (T1)	Mean ± SD Median(IQR)	3.17 ± 1.23 3.00(2.00)	3.70 ± 1.06 4.00(2.00)	−1.12, 0.06	0.461	0.087
	3 months (T2)	Mean ± SD Median(IQR)	3.40 ± 1.22 3.50(1.00)	3.73 ± 1.02 4.00(1.25)	−0.91, 0.25	0.293	0.299
Listening	Baseline (T0)	Mean ± SD Median(IQR)	1.37 ± 0.67 1.00(1.00)	1.30 ± 0.75 1.00(1.00)	−0.30, 0.44	0.098	0.795
	1 month (T1)	Mean ± SD Median(IQR)	1.47 ± 0.57 1.50(1.00)	1.63 ± 0.61 2.00(1.00)	−0.47, 0.15	0.271	0.172
	3 months (T2)	Mean ± SD Median(IQR)	1.50 ± 0.57 2.00(1.00)	1.57 ± 0.68 2.00(1.00)	−0.39, 0.25	0.112	0.452
Total score	Baseline (T0)	Mean ± SD Median(IQR)	9.90 ± 3.41 10.00(5.00)	10.07 ± 3.26 10.50(4.30)	−1.89, 1.55	0.051	0.800
	1 month (T1)	Mean ± SD Median(IQR)	10.10 ± 3.43 11.00(6.00)	12.57 ± 2.87 13.00(4.00)	−4.10, −0.84	0.781	0.002*
	3 months (T2)	Mean ± SD Median(IQR)	11.03 ± 3.189 12.00(4.30)	12.83 ± 2.82 13.50(3.00)	−3.36, −0.24	0.598	0.007*

Within-group comparisons over time revealed significant improvements in Reading (control: *p* = 0.107; intervention: *p* < 0.001), Numeracy (control: *p* = 0.001; intervention: *p* = 0.002), Decision-making (control: *p* = 0.002; intervention: *p* = 0.002), Listening (control: *p* = 0.039; intervention: *p* = 0.001), and Total OHL (control: *p* < 0.001; intervention: *p* < 0.001)

Pairwise post-hoc results:

Reading: Control (–); Intervention (p₁=0.024*, p₂=0.001*, p₃=0.817)

Numeracy: Control (p₁=1.000, p₂=0.099, p₃=0.364); Intervention (*p₁=0.364, p₂=0.364, p₃=1.000*)

Decision-making: Control (*p₁=1.000, p₂=0.526, p₃=0.736);* Intervention (*p₁=0.085, p₂=0.072, p₃ = 1.000*)

Listening: Control (*p₁=1.000, p₂=1.000, p₃=1.000*); Intervention (p*₁=0.467, p₂=0.280, p₃=1.000*)

Total OHL: Control (p₁=1.000, p₂=0.014*, p₃=0.085); Intervention (p₁=0.001*, p₂<0.001*, p₃=0.905)

*Statistically significant differences at p value < 0.05, *p₁*: comparison between baseline and 1 month, *p₂*: comparison between baseline and 3 months, *p₃*: comparison between 1 month and 3 months

Numeracy scores at T0 were comparable between groups (*p =* 0.110), confirmed by small effect size (d = 0.387) and 95% CI crossing zero. The intervention group showed a significant moderate improvement (d = 0.709) at T1 (Intervention:4.00, IQR = 1.00 – Control:3.00, IQR = 2.25; *p =* 0.004) indicating early educational gains. However, the effect size decreased by T2 and the difference was not statistically significant (*p =* 0.095) indicating a smaller clinical impact over time. Within-group comparisons showed significant improvements in both groups (Intervention: *p =* 0.002; Control: *p =* 0.001); however, pairwise comparisons between specific time points were not statistically significant.

Decision-making scores were equivalent at T0 (*p =* 0.581). Improvements in both groups exhibited only small effects and no statistically significant differences at T1 (*p =* 0.087) or T2 (*p =* 0.299). Still, within-group comparisons indicated significant changes over time for both groups (intervention: *p =* 0.002; control: *p =* 0.002), though no pairwise comparisons reached significance.

Listening comprehension scores were similar at T0 (median = 1.00, IQR = 1.00; *p =* 0.795). By T2, both groups showed a median of 2.00 (IQR = 1.00) with only small effects (d = 0.112) and no significant differences between them (*p =* 0.452). Within-group analysis revealed significant changes over time for both groups (intervention: *p =* 0.001; control: *p =* 0.039); however, no specific pairwise differences were found.

Total OHL scores were also similar at T0 (*p* = 0.800). Significant differences emerged between groups at T1 (*p =* 0.002) and T2 (*p =* 0.007), favouring the intervention group, which demonstrated moderate improvements at both time points (d = 0.598 to 0.781). Within-group analysis indicated significant improvement in both groups (*p* < 0.001). Pairwise comparisons in the intervention group showed significant increases from T0 to T1 (*p =* 0.001) and from T0 to T2 (*p* < 0.001), but not between T1 and T2 (*p =* 0.905). In the control group, a significant improvement was only found between T0 and T2 (*p =* 0.014).

### III-Association between PI and OHL scores

A significant negative correlation was found between plaque scores at T2 and both numeracy (rho= − 0.451, *p =* 0.012) and decision-making (rho= − 0.369, *p =* 0.045) within the intervention group (Table 4). This indicates that higher scores in these OHL domains were associated with lower plaque accumulation. No significant correlations were observed for reading (rho= − 0.200, *p =* 0.290), listening (rho= − 0.097, *p =* 0.612), or total OHL scores (rho= − 0.272, *p =* 0.145).

**Table 4:** Correlation between plaque scores after intervention (T2 PI score) and OHL domains in intervention and control groups

OHL domain	Control Group (*n* = 30)		Intervention Group (*n* = 30)	
	rho	*p* value	rho	*p* value
Reading	−0.487	0.006*	−0.200	0.290
Numeracy	−0.052	0.786	−0.451	0.012*
Decision-making	−0.336	0.069	−0.369	0.045*
Listening	0.001	0.994	−0.097	0.612
Total score	−0.421	0.021*	−0.272	0.145

*Statistically significant differences at *p* value < 0.05

In the control group, plaque scores at T2 were significantly and negatively correlated with reading (rho = − 0.487, *p =* 0.006) and total OHL score (rho = − 0.421, *p =* 0.021). No significant associations were found for the other OHL domains (*p* > 0.05).

To explore factors influencing oral health outcomes beyond simple between-group differences, multivariable linear regression analyses were conducted for both PI and OHL at the three-month follow-up. Gender, age, educational level, study group and baseline score were included as covariates based on their association with oral hygiene behaviours [46].

The model for factors affecting PI after three months (Table 5) demonstrated a good overall fit (Adjusted R²=0.556, F = 15.771; *p* < 0.001), with over half of the variance in plaque scores explained by baseline PI score, group assignment, age, gender, and education level. Baseline PI score was the strongest determinant of follow-up PI (B = 0.652, *p* < 0.001), indicating that higher initial plaque levels were associated with higher scores at three months. Group assignment was also a significant predictor (B=−0.460, *p* < 0.001), confirming that participants who received the WhatsApp-based messages had significantly lower plaque scores after three months. Demographic factors, including age, gender, and education, did not show significant associations with PI outcomes.

**Table 5:** Multivariable linear regression for factors affecting PI and OHL after 3 months

Independent Variables	PI			OHL		
	B	(95% CI)	*P* value	B	(95% CI)	*P* value
(Constant)	0.892	(−0.004, 1.788)	0.051	6.263	(2.023, 10.502)	0.005
Age	−0.008	(−0.049, 0.033)	0.707	−0.111	(−0.330, 0.109)	0.316
Study Group						
Intervention	−0.460	(−0.652, −0.268)	< 0.001*	1.668	(0.663, 2.672)	0.002*
Control	Reference					
Gender						
Female	−0.028	(−0.236, 0.181)	0.792	−0.241	(−1.329, 0.846)	0.658
Male	Reference					
Education						
High school/University	0.059	(−0.139, 0.257)	0.554	−0.509	(−1.516, 0.498)	0.316
Primary/Middle school	Reference					
Baseline score	0.652	(0.475, 0.828)	< 0.001*	0.733	(0.580, 0.885)	< 0.001*

*B* regression coefficient, *CI* Confidence Interval

*statistically significant at *p* < 0.05

A similar pattern was observed for OHL, where the model demonstrated a strong fit (Adjusted R²=0.642, F = 22.123; *p* < 0.001), with approximately 67% of the variability in OHL scores explained by the same set of predictors (Table 5). Baseline OHL emerged as the most significant predictor (B = 0.733, *p* < 0.001), where participants with higher literacy levels at baseline tended to maintain or improve their scores after three months. Group assignment also had a statistically significant effect (B = 1.668, *p =* 0.002), highlighting the positive impact of the WhatsApp-based reminders on OHL. Similar to the PI model, age, gender, and education did not show a significant association with OHL at follow-up.

## Discussion

Inadequate oral hygiene during orthodontic treatment can result in the development of WSLs, which may negatively affect both patient outcomes and the reputation of an orthodontic practice. This randomised controlled trial demonstrated that the WHO mOralHealth programme delivered via WhatsApp significantly improved both oral hygiene and OHL among orthodontic patients aged 16–25 years after three months.

The consistent significance of group assignment in both regression models underscores the independent contribution of the intervention beyond baseline measures and demographic characteristics. The positive changes observed in the intervention group highlight the effectiveness of mHealth tools in promoting oral health behaviour, specifically among patients with fixed orthodontic appliances by providing both the knowledge necessary to maintain optimal oral hygiene throughout the course of orthodontic treatment as well as to reinforce behavioural change.

The significant reduction in plaque scores among the intervention group aligns with Pubalan et al. [31] who demonstrated that weekly WhatsApp reminders significantly enhanced oral hygiene among orthodontic patients compared to conventional verbal instruction alone. Similarly, Kumar et al. [47] reported a significant improvement in plaque scores after three months of weekly SMS reminders, consistent with the current findings, where a delayed but significant improvement appeared in oral hygiene by the third month among the intervention group. This pattern supports behavioural psychology models suggesting that habit formation typically requires sustained engagement and consistent reinforcement over time, often taking approximately 66 days [48]. The sustained delivery of WhatsApp messages likely enhanced participants’ self-efficacy and perceived control over their oral health, consistent with Bandura’s Social Cognitive Theory [22], while the gradual reduction in message frequency, as described by the HBM, may have facilitated the shift from externally prompted actions to self-regulated behaviour, reflecting internalisation of the new habits and increased autonomy in maintaining oral hygiene routines. Bowen et al. [49] also observed that automated text message reminders with varying messaging frequencies (12 text messages over four weeks and one text message for eight weeks thereafter) enhanced oral hygiene compliance, while Jones et al. [50] concluded in a systematic review that reminders during orthodontic treatment significantly improved plaque scores, gingival health, and appointment adherence in both short- and long-term follow-up. Similarly, Borujeni et al. [51] found that the use of teledentistry follow-ups during orthodontic treatment significantly improved plaque control and gingival health over the first three visits compared to verbal oral hygiene instructions. Although these studies generally report consistent outcomes, variations in effect sizes may stem from differences in message frequency, age group differences, and interaction modes. Frequent, interactive reminders, especially WhatsApp or app-based interventions, may produce larger effect sizes particularly among younger users with higher motivation and digital engagement.

While most studies confirmed positive trends in intervention groups, the worsening of PI in control group aligns with the findings of Eppright et al. [52] as well as Baherimoghadam et al. [34] where the control group’s oral hygiene deteriorated over time without reminders. The lack of behavioural reinforcement likely contributed to failure in adherence to the given oral hygiene instructions, reflected on increased plaque accumulation. The authors also highlighted that the early phase of orthodontic treatment often presents significant challenges for patients, as they must cope with appliance-related discomfort while simultaneously adopting new oral hygiene practices, which may explain why some studies report no significant improvements in oral hygiene outcomes during the early stages of intervention. Additionally, they noted the influence of a novelty effect; an early improvement in oral hygiene behaviours driven by the initial excitement of a new intervention; which was followed by a regression over time as the novelty diminished [34].

The relationship between OHL and oral hygiene outcomes was demonstrated through domain-specific correlations within each group. The lack of a significant correlation between listening and plaque scores, in both groups, indicates that passive receipt of information alone may not be sufficient to influence behaviour without active comprehension and application [53]. In the intervention group, higher numeracy and decision-making scores were significantly associated with lower plaque scores, while in the control group, reading and total OHL scores were the strongest predictors. Puyén et al. [54] reported that WhatsApp interventions not only improved plaque control but also significantly enhanced knowledge of patients with fixed orthodontics regarding oral hygiene techniques and orthodontic care. These findings align with those of Inegbenosun and Azodo [55] who reported that individuals with low OHL exhibited significantly higher plaque accumulation and gingival inflammation, and Lee et al. [56] who identified OHL and self-efficacy as predictors of dental neglect and preventive oral health behaviours. Those studies support the current finding that higher OHL enhances decision-making and behaviour, supporting the need for targeted interventions to raise OHL as a pathway to improve oral hygiene practices. On the other hand, Sharma et al. [57] found that although SMS improved knowledge and attitudes among mothers, no significant difference in plaque scores was observed between preschoolers in intervention and control groups. Unlike Sharma’s study, the current study targeted orthodontic patients directly affected by plaque accumulation. Consequently, they might have been more motivated to adhere to hygiene protocols when reminded through direct and interactive channels like WhatsApp, which highlights the importance of targeting patients rather than caregivers [57].

Beyond clinical outcomes, this study also demonstrated significant improvements in OHL, especially in reading and numeracy domains, which are essential for interpreting and applying health-related information. These improvements were observed at one month and sustained at three months, agreeing with the findings of Chau et al. [58] who concluded, in a systematic review, that mHealth interventions, including mobile applications and messaging platforms, effectively improved oral health knowledge and behaviours among older adults. Despite targeting a younger population, the appropriately structured, short, digestible message format likely contributed to the observed gains in the current research. Therefore, WhatsApp proved an accessible and engaging platform for delivering oral health information, particularly among adolescents and young adults who are highly engaged with their smartphones. Although some participants actively engaged with the messages, asking questions and seeking clarifications, yet others remained passive readers. Consistent with Chau et al. [58], participants’ preference for personalised content suggests that message customisation may further enhance engagement. Similarly, Mariño et al. [59] demonstrated that participants who received structured web- or app-based oral health content showed improved knowledge, attitudes, and behaviours compared to those receiving conventional information. Similar conclusions were reached by Cardoso et al. [60], who found digital tools effective in promoting OHL in populations with special needs. However, not all digital oral health interventions are equally effective [61, 62], suggesting that intervention design, particularly message frequency, tailored content, and patient interactivity, plays a crucial role in determining effectiveness.

The strength of this study lies in several factors. First, the integration of both objective (PI) and subjective (OHL) measures offered a comprehensive evaluation of knowledge acquisition and behavioural change. Second, the WHO mOralHealth programme used in this study included a series of 46 carefully sequenced messages designed not only to remind but also to educate and motivate individuals, which offered qualitative insights into patient engagement and comprehension that extended beyond quantitative scores. Third, all participants received the same oral hygiene instructions to ensure consistency, and all assessments were performed before patients entered the clinic at each visit, reducing the likelihood that oral hygiene would be temporarily improved by interventions from the orthodontist during the appointment. However, this study has limitations. Although the three-month follow-up period allowed adequate time to capture initial behavioural changes and early habit formation, which might not have been captured with shorter duration studies [61], it remains insufficient to evaluate the durability or long-term sustainability of these effects, which may not last if individuals perceive it as less desirable or if there is no continuous reinforcement [63]. The absence of objective verification of participant engagement with the WhatsApp messages as message delivery and read status do not guarantee that the content was thoroughly viewed or understood. Due to the impossibility of blinding, introduced researcher–participant interaction effects may have influenced responses during follow-up assessments. Social desirability bias introduced by self-reported OHL may have led participants to overestimate their behaviours. The absence of a placebo group restricted the ability to isolate the specific impact of the WhatsApp intervention from general effects related to participant attention or study involvement. Minor measurement errors in the PI cannot be fully excluded; however, all assessments were performed by a single calibrated examiner with verified intra-examiner reliability to ensure consistency. Furthermore, the study’s single-centre design and inclusion of digitally literate orthodontic patients limit generalisability to populations with limited access to mobile technology.

## Conclusion

This study reinforces the effectiveness of mHealth interventions in improving both clinical outcomes (PI) and educational outcomes (OHL), which proved to be a practical approach given the widespread use of WhatsApp among adolescents and young adults. Significant and clinically meaningful improvements were observed in both oral hygiene and OHL, particularly in the Reading domain, which demonstrated large and sustained effects. Numeracy, Decision-making and Listening exhibited smaller effects that are unlikely to be clinically meaningful. The intervention’s consistent effects across demographic groups support the integration of brief, culturally adapted weekly WhatsApp reminders into routine orthodontic practice to enhance patients’ self-care and long-term oral health outcomes, a feasible approach particularly in settings where messages are already used for appointment confirmations, which could be easily adapted to include motivational or educational content aimed at reducing plaque accumulation and its consequences. Tailoring message content to patients’ OHL levels may further enhance patient engagement and oral hygiene compliance. In addition, incorporating such digital interventions into electronic health records could facilitate personalised patient education, progress tracking, and strengthen clinician-patient communication. Future research should explore the optimal frequency of message delivery, the impact of personalised content and the long-term sustainability of behavioural improvements. Moreover, expanding similar interventions across diverse populations with larger sample sizes, longer follow-up periods and a placebo group could further validate and maximise public health impact.

## Data Availability

The data generated and analysed in this study are available upon reasonable request from the corresponding author.

## Appendix I

1. It's important to brush your teeth for 2 minutes with a fluoride toothpaste at least 2 times a day to provide the best protection against tooth decay.
2. Plaque is a sticky film that develops on your teeth. Brushing regular removes this before it hardens and causes damage to your teeth and gums.
3. Try to replace your toothbrush regularly e.g., every three months. A worn-out toothbrush is less effective for cleaning your teeth and removing plaque.
4. Try to brush your teeth with gentle movements. Excessive brushing and too much pressure can damage your teeth and gums.
5. A healthy diet can help prevent tooth decay and gum disease. By reducing your sugar intake, you can lower your risk of gum diseases and tooth decay.
6. It is common to forget to take good care of ourselves when we are stressed. Remember that brushing your teeth is still important during these times.
7. Do you smoke? Not smoking is key for good oral health, fresh-smelling breath, and a healthy smile. Talk to your doctor for support to quit.
8. If you have mouth sores or sensitivity, try brushing with a softer toothbrush.
9. If you want to have a sweet treat, try to have it during a meal rather than as a snack.
10. Enamel is a protective layer on your teeth that can be damaged by sugar in foods and drinks. By eating less sugary food and drinks you can protect your enamel.
11. After brushing spit out the toothpaste foam. You don’t need to rinse your mouth out afterwards because the foam releases fluoride, which strengthens your teeth.
12. Do you forget to brush your teeth? Planning ahead helps. Plan to do it around key events in your day e.g., after taking a shower or right before going to bed.
13. To improve your oral health, try to avoid sugary drinks (sodas, fizzy drinks, energy drinks and fruit juices). Drink water instead!
14. Do you find it hard to remember how long to brush your teeth for? Why not find a 2 min song to play each time you are brushing, or use a timer.
15. Check your mouth regularly at home. If you notice any changes such as red or swollen gums, or any white, dark, or red patches in your mouth - visit a dentist.
16. Did you know that water with added salt is an easy and cheap mouth rinse that keeps your mouth moist and clean?
17. It is more important to brush your teeth regularly than the kind of toothbrush you use. Both manual and electric toothbrushes are fine!
18. Acidic foods and drinks (e.g., lemon) can soften the tooth surface. It is best to wait a bit after eating before brushing your teeth to prevent damage.
19. Prevention is key! Eating a healthy diet and regular brushing will help you maintain good oral health and keep your teeth looking good.
20. Fluoride helps protect your teeth from tooth decay. It stops damage to enamel and repairs it at the same time.
21. Did you know that reducing sugar in your diet is best for your oral health and may reduce your risk of health conditions such as diabetes?
22. After eating or drinking acidic foods (e.g., sugary drinks, oranges, wine) rinse your mouth with water to help reduce acidity and protect your teeth.
23. Did you know that by the age of 21 most people have a full set of 32 permanent teeth, including their wisdom teeth?
24. Good oral hygiene is the best way to protect your mouth from tooth decay and gum disease. Take time to look after your teeth today.
25. It is never too late to start taking care of your gums and teeth. Good oral hygiene at home is essential to keep your mouth healthy and breath smelling fresh.
26. Finding it hard to be motivated to brush your teeth? Think about the positives (e.g., fresh smelling breath). Take a minute to remind yourself of these benefits.
27. Your oral health and your overall health are linked. So, it’s important to take care of your oral health to improve your overall health and wellbeing.
28. Early treatment of tooth decay is important for good oral health and to reduce the risk of pain sensitivity and infection.
29. Taking good care of your oral health and getting early treatment for gum disease can prevent serious damage to your gums and jaw.
30. Taking care of your teeth and eating a balanced diet now will help prevent tooth decay & gum disease as you get older.
31. Taking care of your teeth and mouth is essential for your future health. Good oral health makes you feel good, remain pain-free and have fresh breath.
32. Taking time to brush and care for your teeth while you are young will help to prevent gum disease which can lead to tooth loss in the future.
33. A healthy diet, taking care of your teeth (e.g., brushing, flossing) and regular visits to the dentist help to prevent gum disease (a serious gum infection).
34. Have you brushed your teeth today? Preventing dental plaque build-up helps you prevent gum disease and keeps your teeth healthy.
35. To keep your teeth, gums, and mouth healthy, brush your teeth with fluoride toothpaste for at least 2 min 2 times a day - including before going to bed.
36. Remember to look after your teeth every day to keep your teeth healthy. Setting a daily reminder to brush your teeth on your phone could help.
37. Did you know that the best way to have good oral health is to have good oral hygiene? Use fluoride toothpaste and reduce the amount of sugary food you eat.
38. Try to brush your teeth after meals to protect your teeth from damage caused by the foods you have eaten.
39. Take time to brush your teeth well. Brush for 2 min twice a day to maintain good oral health and keep your breath smelling fresh and your smile healthy.
40. New to cleaning or flossing between your teeth? In the beginning, you may notice some bleeding - be gentle to your gums! Ask a dentist for help if you need it.
41. To clean small spaces between your teeth, use dental floss or tape. If you find it hard to clean your teeth, talk to your dentist for help and support.
42. Worried about bad breath? This could be a sign of gum disease so it’s a good idea to talk to a dentist. Taking good care of your teeth will help.
43. Have you had your teeth checked recently? Having regular check-ups with a dentist is important. Need help finding a dentist? Go to [service link].
44. If you have questions about your teeth or mouth, talk to an oral health professional, e.g., dentist or dental nurse. Go to [add link] for services in your area.
45. Water is your best choice of drink when it comes to protecting your teeth from tooth decay.
46. It is a good idea to visit your dentist regularly for a check-up, even if you have good oral health.

## Abbreviations

WHO: World Health Organisation
HL: Health Literacy
OHL: Oral Health Literacy
WSLs: White Spot Lesions
mHealth: Mobile Health
ITU: International Telecommunication Union
NCDs: Non–Communicable Diseases
RCT: Randomised Controlled Clinical Trial
CONSORT: Consolidated Standards of Reporting Trials
T0: Baseline
T1: 1 month follow–up
T2: 3 months follow–up
PI: Silness–Löe Plaque Index
OHL-AQ: Oral Health Literacy Adult Questionnaire
SPSS: Statistical Package for the Social Sciences
SD: Standard Deviation
IQR: Interquartile Range
CI: Confidence Interval
HBM: Health Belief Model
